# Impact of SNPs on Protein Phosphorylation Status in Rice (*Oryza sativa* L.)

**DOI:** 10.3390/ijms17111738

**Published:** 2016-11-11

**Authors:** Shoukai Lin, Lijuan Chen, Huan Tao, Jian Huang, Chaoqun Xu, Lin Li, Shiwei Ma, Tian Tian, Wei Liu, Lichun Xue, Yufang Ai, Huaqin He

**Affiliations:** 1 College of Life Science, Fujian Agriculture and Forestry University, Fuzhou 350002, China; linshoukai@ptu.edu.cn (S.L.); chenlj1133@gmail.com (L.C.); taoh1620@gmail.com (H.T.); sportsman001@21cn.com (J.H.); 1140561002@fafu.edu.cn (C.X.); linglingdaiguo@gmail.com (L.L.); 1120941001@fafu.edu.cn (S.M.); ttian3860@gmail.com (T.T.); liuw20189@gmail.com (W.L.); 2 College of Environmental and Biological Engineering, Putian University, Putian 351100, China

**Keywords:** rice (*Oryza sativa* L.), single nucleotide polymorphisms (SNPs), protein phosphorylation, impact

## Abstract

Single nucleotide polymorphisms (SNPs) are widely used in functional genomics and genetics research work. The high-quality sequence of rice genome has provided a genome-wide SNP and proteome resource. However, the impact of SNPs on protein phosphorylation status in rice is not fully understood. In this paper, we firstly updated rice SNP resource based on the new rice genome Ver. 7.0, then systematically analyzed the potential impact of Non-synonymous SNPs (nsSNPs) on the protein phosphorylation status. There were 3,897,312 SNPs in Ver. 7.0 rice genome, among which 9.9% was nsSNPs. Whilst, a total 2,508,261 phosphorylated sites were predicted in rice proteome. Interestingly, we observed that 150,197 (39.1%) nsSNPs could influence protein phosphorylation status, among which 52.2% might induce changes of protein kinase (PK) types for adjacent phosphorylation sites. We constructed a database, SNP_rice, to deposit the updated rice SNP resource and phosSNPs information. It was freely available to academic researchers at http://bioinformatics.fafu.edu.cn. As a case study, we detected five nsSNPs that potentially influenced heterotrimeric G proteins phosphorylation status in rice, indicating that genetic polymorphisms showed impact on the signal transduction by influencing the phosphorylation status of heterotrimeric G proteins. The results in this work could be a useful resource for future experimental identification and provide interesting information for better rice breeding.

## 1. Introduction

Rice is one of the most important crops in the world. The draft sequences of two main cultivated rice genomes, *indica* (93–11) and *Japonica* (Nipponbare), were all reported in 2002 [1,2]. After that, the Rice Annotation Project Database (RAP-DB) (http://rapdb.dna.affrc.go.jp/) and the Michigan State University (MSU) Rice Genome Annotation Project (http://rice.plantbiology.msu.edu/) both provided high-quality and timely annotation for the Nipponbare reference genome [3]. With the development of high-throughput sequencing methods, more and more rice genotypes have been resequenced in recent years [4,5]. This will provide abundant information on the genetic variations of different rice genotype individuals, including copy number variations (CNVs) and single nucleotide polymorphisms (SNPs).

SNPs are DNA sequence variations occurring when a single nucleotide in the genome differs between members of a biological species [6]. SNPs might occur in different regions related to transcription and translation, including gene coding region, introns, untranslated regions (UTRs), promoter regions and intergenic regions. Non-synonymous SNPs (nsSNPs) located in the gene coding regions change the coding amino acids of protein sequences. In human beings, the researchers found that 90% of genetic variations are caused by SNPs [7]. In rice (*Oryza sativa* L.), SNP mutations causing protein-coding changes or gene expression alterations both have the potential to account for rice agronomic traits [8,9]. In addition, a larger proportion of mutations involved in crop improvement are protein altering rather than regulatory changes [9,10].

Non-synonymous SNPs that cause coding amino acid change would have the potential to influence protein phosphorylation status [11,12]. Erxleben et al. first used the term “phosphorylopathy” to describe genetic variation that results in inaberrant regulation of protein phosphorylation [13]. In 2010, Ren et al. defined an nsSNP that affected the protein phosphorylation status as a phosphorylation-related SNP (phosSNP) [14]. Ryu et al. carried out a large scale survey of potential phosphovariants in humans, which were defined as amino acid variations that might influence protein phosphorylation status [12]. Ren et al. performed a genome-wide analysis of genetic polymorphisms that influence protein phosphorylation in humans [14]. However, to our best knowledge, the potential impact of SNPs on the protein phosphorylation status in rice is not clearly understood. In plants, phosphorylation is one of the most important post-translational modifications (PTMs) of proteins that have essential roles in the majority of biological pathways, regulating cellular processes like metabolism, proliferation, differentiation and apoptosis [15]. A large number of phosphorylation sites in rice had been identified by Nakagami et al. [16]. Moreover, a rice-specific phosphorylation site predictor, Rice_phospho 1.0, had been developed [15]. The resources of SNPs and phosphorylation sites in rice genome and proteome could contribute to the comprehensive studies of the impact of SNPs on protein phosphorylation status.

In this paper, we performed a genome-wide analysis of SNPs that potentially impacted the protein phosphorylation status in rice. Firstly, we updated the rice SNPs resource based on the new rice genome data (Ver. 7.0) and predicted protein phosphorylation sites in rice by using NetPhosK 1.0 and Rice_phospho 1.0. After that, these two data were integrated to analyze the relationship between SNPs and protein phosphorylation sites. Finally, using heterotrimeric G protein as a case study, we interpreted the impact of nsSNP on phosphorylation sites and the function of heterotrimeric G protein in rice.

## 2. Results

### 2.1. SNPs in Rice Genome Ver. 4.0 and 7.0

**Figure 1:**
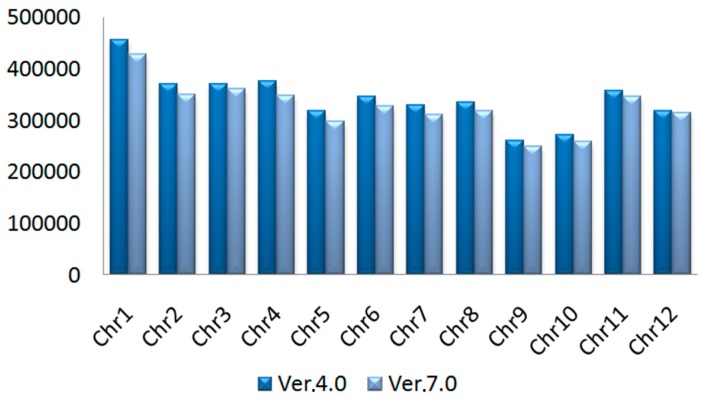
Single nucleotide polymorphisms (SNPs) in different chromosomes in rice genome 4.0 and 7.0. Chr mean chromosome. The same as below.

First, the data of 4,109,378 SNPs in rice genome Ver. 4.0 were downloaded from http://www.ncgr.ac.cn/RiceHap2. Then, they were mapped to rice genome Ver. 7.0 by BLASTn. Finally, a total 3,907,374 SNPs were detected in rice genome Ver. 7.0, which was lower than that in rice genome Ver. 4.0 (Figure 1). This result indicated that the redundancy of SNPs in rice genome had been removed.

### 2.2. nsSNPs in Rice Genome Ver. 7.0

The different SNPs in rice genome Ver. 7.0 were classified into different types based on SNP location, which were shown in Table 1. It could be found that most of the SNPs were located in the Inter-gene region, UTR region and Intron region. There were 314,228 synonymous SNPs and 384,565ns SNPs in rice genome. Whilst, nsSNPs accounted for 9.9% of total SNPs in rice genome Ver. 7.0, which could change the amino acids in 48,961 proteins. The nsSNPs that result in Premature Termination Codons (PTCs) were then removed from the nsSNPs dataset which was used for subsequent analysis.

### 2.3. Prediction of PhosSNPs in Rice Genome

NetPhosK 1.0, a kinase-specific phosphorylation site predictor, was used to predict potential kinase-specific phosphorylation sites in rice proteome and in the corresponding variant sequences induced by nsSNPs, respectively. The results were confirmed by using Rice_phospho1.0. The common phosphorylation sites predicted by NetPhosK 1.0 and Rice_phospho1.0 were employed in the following research work. A total of 2,508,261 potential phosphorylation sites were achieved in the rice proteome. According to the definition of phosSNPs and different types of phosSNPs [12,14], we wrote a PERL script to identify phosSNPs among the predicted phosphorylation sites in rice proteome. The results were shown in Table 2. We found that 39.06% nsSNPs in rice genome were phosSNPs, among which there were 25,511 Type I, 14,615 Type II, 78,365 Type III and 31,706 Type IV phosSNPs (Table 2). A nsSNP to create or remove a phosphorylation site was named Type I (+) or Type I (−) phosSNP. The Type I phosSNP took up 16.99% of total phosSNPs. There were only 11 Type I (+) phosSNPs in rice genome, while others were Type I (−) phosSNPs. A nsSNP to create or remove adjacent phosphorylation sites was termed Type II (+) or Type II (−) phosSNP. Type II phosSNPs just occupied 9.73% of total phosSNPs and all of them were Type II (−) phosSNPs. A nsSNPs to induce changes of PK types in adjacent phosphorylation sites was Type III phosSNP. The Type III phosSNPs were the most predominant phosSNPs type and accounted for 52.17% of total phosSNPs in rice genome. nsSNPs were shown to cause an amino acid substitution among Ser, Thr, or Tyr, thus Type IV phosSNP induced a change of PK types for the phosphorylation site. Type IV phosSNPs took up 21.11% of total phosSNPs.

**Figure 2:**
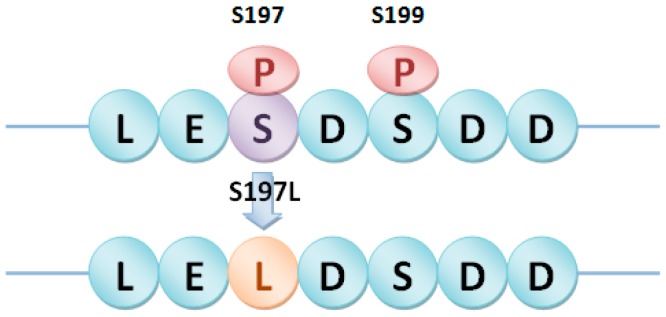
Type I (−) and Type II (−) phosSNP S197L in LOC_Os05g11370 removed the experimentally identified protein phosphorylation sites S197 and S199. Blue circle: Amino acid; Purple circle: Amino acid before mutation; Orange circle: Amino acid after mutation caused by phosSNP; Red oval with “P”: Phosphate group; The same as below.

Furthermore, the experimentally identified phosphorylation sites in rice, which were collected in our previous research work [15], were also used to detect the potential phosSNPs. Due to the limited information of PK specific for rice protein phosphorylation sites, we only predicted Type I and II phosSNPs in the identified rice phosphorylation sites. In total, 41 Type I and 85 Type II phosSNPs were predicted in 97 proteins. For example, phosSNP S197L, located in a conversed unknown protein LOC_Os05g11370, could be defined as both Type I (−) and Type II (−) phosSNP because it could remove the experimentally identified phosphorylation sites of S197 and S199 (Figure 2).

### 2.4. PhosSNPs in Heterotrimeric G Proteins

**Figure 3:**
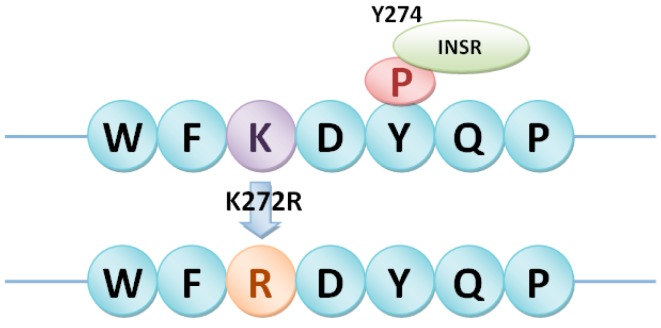
Type II (−) phosSNP, K272R, in heterotrimeric Gα subunit (LOC_Os05g26890) removed the adjacent protein phosphorylation site of Y274. Green oval: Specific kinase type of the phosphorylation site; The same as below. INSR: Insulin receptor tyrosine kinase.

**Figure 4:**
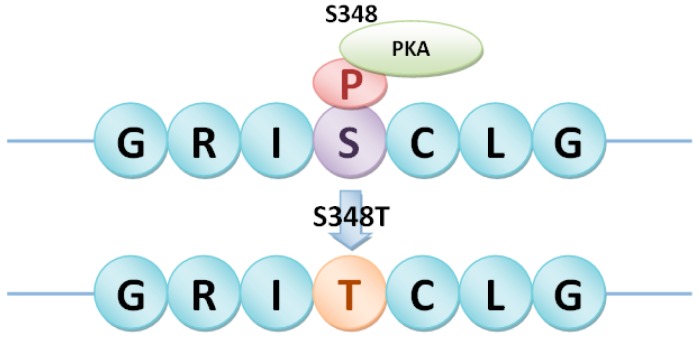
Type I (−) phosSNP, S348T, in heterotrimeric Gβ subunit (LOC_Os03g46650) removed the protein phosphorylation site S348. PKA: Protein kinase A.

**Figure 5:**
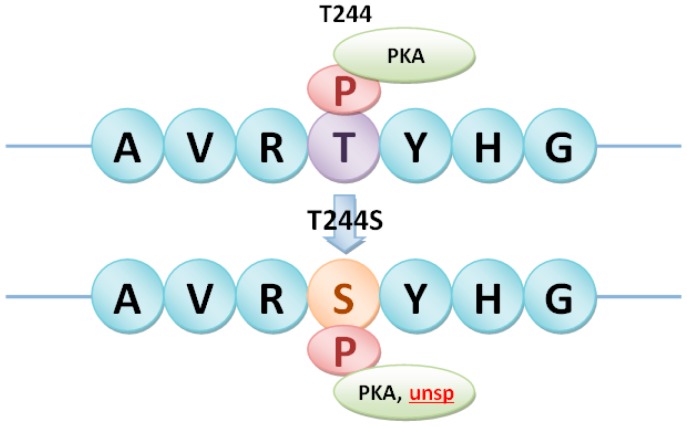
Type IV phosSNP, T244S, in heterotrimeric Gβ subunit (LOC_Os03g46650) induced the substitution between Thr and Ser in the protein phosphorylation site T244 and changed kinase types of the target site. The red marks represented the different kinase types caused by phosSNPs; The same as below. PKA: Protein kinase A; unsp: non-specific prediction kinase.

**Figure 6:**
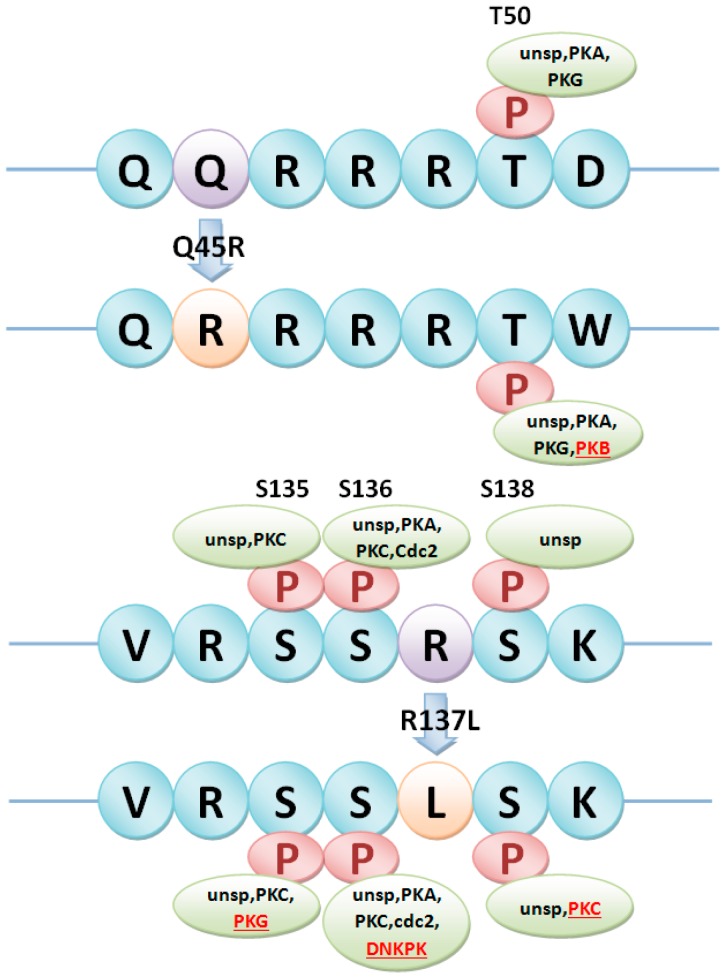
Two Type III phosSNPs, Q45R and R137L, in heterotrimericGγ2 subunit (LOC_Os02g04520) changed the kinase types in the adjacent protein phosphorylation sites of T50, S135, S136 and S138. PKA: Protein kinase A; PKB: Protein kinase B; PKC: Protein kinase C; PKG: Protein kinase G; Cdc2: Cell division cycle 2 kinase; DNKPK: DNA-Dependent Protein Kinase; unsp: non-specific prediction kinase.

Heterotrimeric G proteins in rice were then selected as a case study. There were 1, 4 and 2nsSNPs in Gα subunit (LOC_Os05g26890), Gβ subunit (LOC_Os03g46650) and Gγ2 subunit (LOC_Os02g04520), respectively (Table 3). The relationship between the predicted phosphorylation sites and nsSNPs in heterotrimeric G proteins was analyzed to identify phosSNPs. As shown in Table 3, five nsSNPs in heterotrimeric G proteins were phosSNPs, including K272R in Gα, T244S and S348T in Gβ, Q45R and R137L in Gγ2. These phosSNPs were assigned to Type I (−), Type II (−), Type III and Type IV, which were shown in Figure 3, Figure 4, Figure 5 and Figure 6.

In Figure 3, we found that K272RnsSNP in Gα subunit (LOC_Os05g26890) of heterotrimeric G proteins in rice was a Type II (−) phosSNP. Because Gα subunit harbored the K272RnsSNP to cause its nearby phosphorylation site Y-274 to be dephosphorylated. Of course, we will carry out a further experimental identification to detect whether the Tyr-274 site is really not phosphorylated in the K272R allele. As shown in Figure 4, S348T in Gβ subunit (LOC_Os03g46650) of heterotrimeric G protein in rice was predicted as a Type I (−) phosSNP. Gβ subunit was potentially phosphorylated by protein kinase A (PKA) at Ser-348. However, the S348T nsSNP might remove the phosphorylation site (Figure 4). Meanwhile, the Gβ subunit was also predicted to be phosphorylated at Thr-244, whereas the T244SnsSNP might change the PK types at position 244. Therefore, T244S nsSNP of Gβ subunit was regarded as a Type IV phosSNP (Figure 5). Interestingly, the Gβ subunit was experimentally identified to be phosphorylated at serine-246 sites [17]. The above results showed that Type III phosSNPs were the most predominant phosSNPs type in rice genome. We found two Type III phosSNPs, Q45R and R137L, in Gγ2 subunit (LOC_Os02g04520) of heterotrimeric G proteins in rice. The Q45R nsSNP of Gγ2 subunit could alter the PK types for T50, while R137L might change the PK types for Ser-135, Ser-136 and Ser-138 (Figure 6). In summary, the prediction results were not only consistent with previous experimental studies but also provided a useful resource for further experimental identification.

## 3. Discussion

A genome-wide SNP resource was comprised of 4.11 million loci polymorphism between the two major cultivated rice subspecies, *indica* (9311) and *japonica* (Nipponbare) [6,18]. This SNP resource is freely accessible at RiceHap2 and the National Center for Biotechnology Information (NCBI) SNP database (NCBI dbSNP build 132) as “reference SNPs (rsSNPs)” with detailed annotations on the rice genome. However, efforts to improve the quality of rice SNP resources are limited, which is affecting large-scale genotyping applications of this important crop [19]. With the development of high-quality assemblies of rice genome [4], SNP resources for rice genome should be updated. By using the updated rice genome Ver. 7.0, we confirmed 3.90 million loci polymorphic between *indica* (9311) and *japonica* (Nipponbare). We found only 9.9% of SNPs were nsSNPs. Ren et al. indicated that a very small proportion of human SNPs were nsSNPs (<1%) [14]. Finally, we constructed a database, SNP_rice, to deposit the new version of the SNP resource between *indica* (9311) and *japonica* (Nipponbare), which could be accessed at http://bioinformatics.fafu.edu.cn/SNP-rice. 

Protein phosphorylation plays essential roles in the majority of biological pathways. Genome-wide prediction of phosphorylopathies in rice might provide a highly valuable resource for further experimental identifications. In this research work, we predicted 2,508,261 phosphorylation sites in rice proteome by using the combination of NetPhosK1.0 and Rice_phospho 1.0. After conducting a systematic analysis, we observed that 150,197 (39.1%) nsSNPs could affect protein phosphorylation. In particular, 52.2% of phosSNPs were Type III phosSNPs, which induced changes of PK types for adjacent phosphorylation sites rather than creating or removing phosphorylation sites. This was consistent with the results in the research work of Ren et al. [14]. They found that 78.8% of phosSNPs in humans were Type III phosSNPs, which was more than the other types of phosSNPs. In this regard, most nsSNPs might regulate protein phosphorylation dynamics and play ubiquitous roles in rewiring the biological pathways. We also integrated the rice phosSNPs information into the above database, SNP_rice, which was freely available for academic researchers. The results in this work could be a useful resource for future experimental identification.

Heterotrimeric G protein signaling cascades is one of the primary sensing mechanisms between the cell and environment in metazoans. In our previous research work, we detected heterotrimeric G-protein subunits in rice were phosphorylated to transduct abscisic acid (ABA) and drought stress signal [17,20]. Our research results were consistent with Nakagami et al. (2010) and Aranda-Sicilia et al. (2015) [16,21]. They also found heterotrimeric G protein subunits were phosphorylated in vivo. Thus, the previous results indicated that phosphorylation of heterotrimeric G protein subunits in rice was likely to be important in the signal transduction. A new publication reported by Trusov and Botella proposed that, instead of the guanosine triphosphate/guanosine diphosphate (GTP/GDP) cycle used in animals, plant heterotrimeric G protein under phosphorylation status were activated to transduct the signals between the cell and the environment [22]. In this work, we found five nsSNPs that potentially influenced heterotrimeric G protein phosphorylation status. The experiments in the laboratory by Trusov and Botella seemed to indicate that substitution of several of the phosphorylated residues with non-phosphorylatable residues renders the subunits inactive and thus unable to restore a wild type phenotype in their respective Arabidopsis mutants [22]. Therefore, we could conclude here that genetic polymorphisms in heterotrimeric G protein had an impact on their phosphorylation status and thus influenced signal transduction.

## 4. Materials and Methods

### 4.1. SNPs in Rice Genome 4.0 and 7.0

SNPs data were downloaded from RiceHap2 database (http://www.ncgr.ac.cn/RiceHap2/index.html), while DNA sequences of rice genome Ver. 4.0 (IRGSP Build 4.0) and 7.0 (MSU Release 7.0) were downloaded from RAP-DB (http://rapdb.dna.affrc.go.jp/download/build4.html) and MSU-RAP (ftp://ftp.plantbiology.msu.edu/pub/data/Eukaryotic_Projects/o_sativa/annotation_dbs/pseudomolecules/), respectively. SNPs data were first subjected to rice genome Ver. 4.0 to get the DNA sequences including SNPs and then mapped to rice genome Ver. 7.0 to create SNPs in the new version rice genome. Meanwhile, the rice genome Ver. 7.0 annotation files, including allseq, cds (coding sequences) and all gff3, were downloaded.

Based on the location, SNPs in rice genome 7.0 were classified into 5 groups, Inter-gene SNPs, UTR SNPs, Intron SNPs and Coding region SNPs (cSNP). The cSNPs were further grouped into non-synonymous SNPs (nsSNPs) or synonymous SNP (sSNPs), which was defined according to whether a cSNP could cause an amino acid substitution or not. Then, sSNPs that result in Premature Termination Codons (PTCs) were removed according to Ren et al. [14].

### 4.2. Prediction of Phosphorylation Sites in Rice Proteome

First, we took the protein sequence in rice genome Ver. 7.0 as benchmark sequence data. Then, we made changes to a protein sequence, one of its nsSNPs at a time, to prepare a variant sequence. NetPhosK 1.0 (http://www.cbs.dtu.dk/services/NetPhosK/) was used to scan the benchmark protein sequences and variant protein sequences for the phosphorylation sites and specific kinases with the high threshold, respectively. The predicting phosphorylation sites were confirmed by using Rice_phospho1.0 (http://bioinformatics.fafu.edu.cn), which was a rice-specific phosphorylation sites predictor.

The 15-mer sequences of phosphorylation sites, P (−7, +7), which was defined as a phospho-Ser, phospho-Thr, or phospho-Tyr flanked by 7 residues upstream and 7 residues downstream, were extracted from the protein sequences and constructed as a dataset. By comparing results of the two kinds of phosphorylation site datasets, the phosSNP that might influence protein phosphorylation status could be detected based on the definition.

### 4.3. Detection of Different Types of phosSNPs

According to the definitions given by Ren et al. and Ryu et al. [12,14], we detected different types of phosSNPs in the above results, including Type I (+)/(−), Type II (+)/(−), Type III and Type IV phosSNPs. An sSNP inducing mutation of an amino acid with Ser/Thr/Tyr residue to create a potential new phosphorylation site was named Type I (+) phosSNP, while the vice versa to remove an original phosphorylation site was named Type I (−) phosSNP. A nsSNP to create or remove adjacent phosphorylation sites was termed Type II (+) or Type II(−) phosSNP, and to induce changes of PK types in adjacent phosphorylation sites was Type III phosSNP. Also, the Type IV phosSNP was defined as an amino acid substitution among Ser, Thr, or Tyr that induces a change of PK types for the phosphorylation site; i.e. the target site might still be phosphorylated but by a different type of kinase.

### 4.4. PhosSNPs in Heterotrimeric G Proteins

We downloaded the heterotrimeric G protein sequences from NCBI and searched SNPs in the G protein sequences against the rice genome Ver. 7.0 SNP dataset. We also predicted phosphorylation sites in G protein sequences by using NetPhosK 1.0 and Rice_phospho1.0 tools, then identified different phosSNPs in the heterotrimeric G proteins according to the above protocol.

### 4.5. Database Construction

We used the JAVA to develop the database, SNP-rice 1.0, which was accessible at http://bioinformatics.fafu.edu.cn. The database will be continuously updated when new phosphorylation sites and other SNP data become available.

## 5. Conclusions

In this paper, we attained an updated rice SNP resource, among which 9.9% of SNPs were nsSNPs. Whilst, we observed that 150,197 (39.1%) nsSNPs could potentially influence protein phosphorylation status, among which 52.2% might induce changes of protein kinase (PK) types for adjacent phosphorylation sites. Finally, we constructed a database, SNP_rice, to deposit the updated rice SNP resource and phosSNPs information, which could be freely available for academic researchers at http://bioinformatics.fafu.edu.cn. As a case study, we detected five nsSNPs that potentially influenced heterotrimeric G protein phosphorylation status in rice, indicating that genetic polymorphisms had an impact on the signal transduction between the cell and the environment by influencing the phosphorylation status of heterotrimeric G proteins.

## Figures and Tables

**Table 1:** Different SNPs on different chromosomes in rice genome 7.0.

Chromosome	Inter-Gene	Intron	UTR	sSNP	nsSNP	Number of Proteins Holding SNPs
Chr1	260,587	80,970	18,498	32,343	35,815	5709
Chr2	212,543	65,908	14,728	24,573	32,379	4693
Chr3	213,338	66,415	15,408	36,203	28,847	4771
Chr4	207,656	61,928	11,237	31,795	34,520	4531
Chr5	181,707	51,407	10,337	21,797	32,094	4034
Chr6	202,075	58,375	10,506	22,845	33,679	4190
Chr7	190,062	54,368	10,492	25,042	30,964	3884
Chr8	194,303	56,258	10,264	22,845	33,969	3779
Chr9	151,668	43,941	7336	18,703	26,741	3031
Chr10	161,059	43,460	7593	22,062	25,383	3024
Chr11	210,082	60,511	9046	31,228	34,399	3716
Chr12	193,270	52,636	8547	24,792	35,775	3599
Total	2,378,350	696,177	133,992	314,228	384,565	48,961

**Table 2:** Different phosSNPs on different chromosomes in rice genome 7.0.

Chromosome	Type I (−)	Type I (+)	Type II (−)	Type III	Type IV
Chr1	2803	0	1411	7996	3215
Chr2	2608	1	1374	6778	2693
Chr3	2065	3	1034	6004	2491
Chr4	2473	0	1284	6296	2513
Chr5	2900	1	1466	6844	2878
Chr6	3015	0	1348	7528	2852
Chr7	2351	0	1028	6155	2455
Chr8	3054	2	1491	7174	2949
Chr9	2170	1	921	5299	2123
Chr10	1805	2	910	4842	1916
Chr11	2423	0	1021	6347	2630
Chr12	3238	1	1327	7102	2991
Total	25,500	11	14,615	78,365	31,706

**Table 3:** nsSNPs in heterotrimeric G proteins in rice.

Subunits	LOC ID	nsSNP ID	Nucleotide Mutation	Animo Acid Mutation
Gα	LOC_Os05g26890	SNP050177186	T/C	K272R
Gβ	LOC_Os03g46650	SNP030274451	A/G	S348T
		SNP030274452	T/C	R279G
		SNP030274453	T/A	T244S
		SNP030274454	A/G	N217T
Gγ2	LOC_Os02g04520	SNP020015843	A/G	Q45R
		SNP020015851	G/T	R137L
